# Influence of fermentation conditions on the surface properties and adhesion of *Lactobacillus rhamnosus* GG

**DOI:** 10.1186/1475-2859-11-116

**Published:** 2012-08-29

**Authors:** Gurjot Deepika, Esther Karunakaran, Claire R Hurley, Catherine A Biggs, Dimitris Charalampopoulos

**Affiliations:** 1 Food and Nutritional Sciences, University of Reading, PO Box 226, Reading, UK, RG6 6AP; 2 CheLSI Institute, Chemical and Biological Engineering, University of Sheffield, Sheffield, UK; 3 Sheffield Surface Analysis Centre, Kroto Research Institute, University of Sheffield, Sheffield, UK

## Abstract

**Background:**

The surface properties of probiotic bacteria influence to a large extent their interactions within the gut ecosystem. There is limited amount of information on the effect of the production process on the surface properties of probiotic lactobacilli in relation to the mechanisms of their adhesion to the gastrointestinal mucosa. The aim of this work was to investigate the effect of the fermentation pH and temperature on the surface properties and adhesion ability to Caco-2 cells of the probiotic strain *Lactobacillus rhamnosus* GG.

**Results:**

The cells were grown at pH 5, 5.5, 6 (temperature 37°C) and at pH 6.5 (temperature 25°C, 30°C and 37°C), and their surfaces analysed by X-ray photoelectron spectrometry (XPS), Fourier transform infrared spectroscopy (FT-IR) and gel-based proteomics. The results indicated that for all the fermentation conditions, with the exception of pH 5, a higher nitrogen to carbon ratio and a lower phosphate content was observed at the surface of the bacteria, which resulted in a lower surface hydrophobicity and reduced adhesion levels to Caco-2 cells as compared to the control fermentation (pH 6.5, 37°C). A number of adhesive proteins, which have been suggested in previous published works to take part in the adhesion of bacteria to the human gastrointestinal tract, were identified by proteomic analysis, with no significant differences between samples however.

**Conclusions:**

The temperature and the pH of the fermentation influenced the surface composition, hydrophobicity and the levels of adhesion of *L. rhamnosus* GG to Caco-2 cells. It was deduced from the data that a protein rich surface reduced the adhesion ability of the cells.

## Introduction

Lactic acid bacteria and in particular lactobacilli have been extensively used in the food and pharmaceutical industries and play an important role in the control of undesirable microorganisms in the intestinal and urogenital tract. For this reason, their use as probiotics has been extensively studied, aiming to elucidate the mechanisms of actions and produce strains with enhanced activities. One of the main criteria for selecting probiotic strains is their ability to adhere to the intestinal epithelium, as this determines their interactions with the host and the microorganisms present in the host system [1]. The adhesion of probiotic bacteria to the gastrointestinal tract is commonly tested *in vitro* using model cell lines, such as Caco-2 cells [2], mucus [3] and extracellular matrix components, such as collagens, fibronectin and laminin [4]. Certain studies have suggested a correlation between *in vitro* and *in vivo* results, indicating that *in vitro* adhesion could be used for predicting the residence time of probiotics in the human gastrointestinal tract [5,6]. The adhesion ability of probiotics is closely linked with their surface properties, as these influence to a large extent the interactions within the gut ecosystem. The cell wall of Gram positive bacteria, including that of lactobacilli, consists of a thick peptidoglycan layer, which is decorated with various components, including (lipo-) teichoic acids, polysaccharides, covalently bound proteins and S-layer proteins [7,8].

In general, the production process for probiotics involves the batch growth of probiotics in large scale bioreactors. Research so far has shown that the fermentation characteristics, including the growth medium and the fermentation temperature and pH, besides affecting the cell yield, can also affect the technological properties of the cells. These include for example their ability to survive freezing or freeze drying and storage post-drying, or survive in highly acidic solutions or in the presence of high bile concentrations [9-12]. It has been suggested that these properties are influenced by the physiological state of the cells, and are associated with changes in the fatty acid composition, the membrane permeability or the enzymatic activities of the cells [12,13]. Although there is a good indication of the link that exists between the production process and the physiological characteristics of the cells, there is a limited knowledge on the effect of the production process on the surface properties of probiotic lactobacilli, and on how these relate to the adhesion of the probiotic cells to the gastrointestinal mucosa. The factors that have been investigated so far, and which have been shown to influence bacterial adhesion to Caco-2 cells, include the incubation time and the composition of the growth medium [14,15]. Moreover, research from our group using *Lactobacillus rhamnosus* GG, a well-established probiotic strain, indicated that the adhesion of the bacterial cells to Caco-2 cells is influenced by the presence of proteins and non-proteinaceous compounds, such as carbohydrates and phosphate-containing compounds on the surface of the cells, and is affected by the growth time [15]. The aim of this work was to investigate the effect of the fermentation pH (pH 5, 6, 6.5 and uncontrolled pH) and temperature (25, 30 and 37°C, all at pH 6.5) on the surface properties of *L. rhamnosus* GG and on its ability to adhere to Caco-2 cells. The rationale behind this is that sub-optimal process conditions have been shown to affect the technological properties of the cells and are therefore likely that they can affect the surface properties of the cells, and consequently their adhesion abilities. This work is novel and important in our effort to understand the adhesion mechanisms and identify methodologies to manipulate the adhesion abilities of the strains. To this end, a variety of spectroscopic techniques and gel based proteomic analysis were employed to study the surface composition of the bacterial cells.

## Materials and methods

### Bacterial strain and growth conditions

*Lactobacillus rhamnosus* GG (ATCC 53103) was obtained from ATCC (Middlesex, UK) and was stored at −80°C in 2 ml cryovials containing 20% v v^-1^ glycerol. The inoculum was grown overnight in 10 ml de Man Rogosa Sharpe (MRS) medium (Oxoid, UK) at 37°C. The cells from the overnight culture were washed with phosphate buffered saline (PBS) (Oxoid, UK) to remove the carryover medium and then re-suspended in PBS. An aliquot from the cell suspension was used to inoculate the fermentation medium so that an initial optical density (OD_600_) of 0.2 was obtained. The fermentation medium consisted of 20 g l^-1^ glucose (Sigma, Poole, UK), 10 g l^-1^ yeast extract (Oxoid, Basingstoke, UK), 15 g l^-1^ vegetable peptone (Oxoid), 1% Tween 80 (Sigma, Poole, UK), 0.2 g l^-1^ MgSO_4_ × 7H_2_O (VWR, Lutterworth, UK), 0.05 g l^-1^ MnSO4 × 4H_2_O (VWR), and 0.1 M phosphate buffer (VWR). The fermentations were carried out anaerobically, with continuous addition of N_2_, in 300 ml custom-made glass bioreactors containing 200 ml of media, at different conditions. These included various pH values, i.e. pH 6.5, pH 6, pH 5.5, pH 5 and uncontrolled pH (all at a temperature of 37°C), and temperatures, i.e. 25°C, 30°C, and 37°C (all at a pH of 6.5). The control fermentation was carried out at 37°C, with the pH at 6.5. The growth of the bacteria was monitored by measuring the OD_600_ and by viable cell counting using MRS agar (Oxoid, UK) plates. The fermentations were performed in duplicate. The cultures were incubated for up to 2 h after the end of the stationary phase, and the bacteria harvested by centrifugation (3000 g) and freeze dried in 10% sucrose (VWR) solution, as described previously [14]. The cells were collected by centrifugation at 3000 g for 10 min and re-suspended in 5 ml of 10% (w v^-1^) sucrose solution to obtain an OD_600_ value of about 4. The cell suspension was frozen at −80°C and then dried in an IEC Lyoprep-3000 freeze dryer (Lyoprep, Dunstable, UK). The freeze dried cells were stored at room temperature in desiccators for further analysis.

### X-ray photoelectron spectroscopy (XPS)

For XPS analysis, the freeze dried bacterial samples were washed 3 times with sterile ultra-pure water and the pellets were aseptically air dried on glass cover slips. These were then mounted onto the standard sample bar using double sided adhesive tape. The XPS measurements were made with a KRATOS AXIS Ultra DLD Photoelectron spectrometer at 10 kV and 15 mA, using an Al Kα X-ray source (1486.6 eV) based on a previously described method [15]. The takeoff angle was fixed at 90°. For each sample, the data were collected from three randomly selected locations, and the sampling area was 300 × 700 *μ*m. The analysis consisted of a wide survey scan (160 eV pass energy; 1.0 eV step size) and a high-resolution scan (20 eV pass energy; 0.1 eV step size). The binding energies of the peaks were determined using the C1s peak at 284.6 eV. The software Casa XPS 2.3.1250 was used to fit the XPS spectra peaks. No constraint was applied to the initial binding energy values, however the full width at half-maximum (FWHM) was maintained constant for the carbon contributions in a particular spectrum.

### Fourier transform infrared spectroscopy (FT-IR)

Fourier transform infrared spectroscopy (FT-IR) was performed using an IR Prestige-21 (Shimadzu) FT-IR spectrophotometer equipped with an attenuated total reflectance (ATR) system (Pike Technologies) as previously described [14]. To prepare the sample, the freeze-dried bacteria were re-suspended in 1 ml of sterile deionised (DI) water; 20 μl of this cell suspension were allowed to air-dry on a diamond crystal attached to the spectrophotometer. At least 64 scans between 4000 and 900 cm^-1^ with a resolution of 4 cm^1^ were recorded for each sample using the Happ-Genzel apodisation function. Principal component analysis (PCA) of the FTIR spectra was carried out at the wave number region 800–1800 cm^-1^with XLSTAT software (http://www.xlstat.com/; version 13.1.05) using the Pearson correlation.

### Proteomic analysis

#### Extraction and SDS PAGE analysis of cell wall associated proteins

The cell wall associated proteins were extracted as described previously, with some modifications [16]. Briefly, the freeze dried bacteria were washed and the cell pellet, corresponding to 20 ODml of culture (ODml is defined as the OD_600_ of the cell suspension multiplied by the volume of the suspension, in ml), was re-suspended in 3 ml of extraction buffer (100 mM Tris–HCl, pH 8.0, 5 mM EDTA and 1 mg ml^-1^ lysozyme), and incubated at 37°C for 3 h. The supernatant was recovered and the proteins were precipitated with ice-cold acetone. The protein pellet was solubilised in loading buffer, and the proteins separated by sodium dodecyl sulphate poly acrylamide gel electrophoresis (SDS-PAGE) using a 12% (w/v) polyacrylamide gel, following the method previously described by Laemmli (1970) [17]. The electrophoresis was carried out at constant voltage (120 V) until the bromophenol blue tracking dye front reached the bottom of the gel. The gels were stained with Bio-Safe Coomassie blue stain (Bio-Rad) according to the manufacturer’s protocol. Low molecular weight protein markers (New England Biolabs) were used as protein standards.

#### In-gel digestion of proteins using trypsin

The protein bands obtained after one dimensional gel electrophoresis were cut out, and the proteins embedded in the gel matrix were digested using trypsin [18]. Briefly, the Coomassie stain was washed away using ammonium bicarbonate and the proteins were reduced using dithiothreitol (DTT) and alkylated using iodoacetamide (IAA). The proteins in the gel matrix were subject to trypsin digestion overnight in the presence of ammonium bicarbonate and acetonitrile. The peptides were eluted from the gel matrix using repeated washing using formic acid, ammonium bicarbonate and acetonitrile, dried using a vacuum concentrator (Eppendorf), and stored at −20°C.

#### Protein identification

The dried peptide samples were re-suspended in 20 μl Switchos buffer (3% acetonitrile and 0.1% trifluoroacetic acid). Mass spectrometry was performed on the samples using an electrospray ionisation (ESI)-ion trap (HCT Ultra, Bruker Daltonics) coupled with an online capillary liquid chromatography system (Famos, Switchos and Ultimate from Dionex/LC Packings). The peptides were separated on a PepMap C-18 RP capillary column (Dionex/LC Packings) at a constant flow rate of 0.3 μl min^-1^, with a linear gradient elution using buffer A (3% acetonitrile and 0.1% formic acid) and buffer B (97% acetonitrile and 0.1% formic acid), starting with 3% buffer A up to 35% buffer B over 45 minutes. Data acquisition was set in the positive ion mode with a mass range of 300 – 2000 m/z. Tandem mass spectrometry was performed on peptides with +2, +3, and +4 charge states.

The identification of the proteins was performed using the *L. rhamnosus* GG ATCC 53103 protein database, downloaded from Uniprot (http://www.uniprot.org). The search parameters were set at a mass tolerance of 1.2 Da, MS/MS tolerance of 0.6 Da, one missed cleavage of trypsin, oxidation of methionine, and cysteine modification with IAA. Molecular Weight Search (MOWSE) scores greater than 50, were considered significant [19]. The hydrophobicity of the identified proteins was determined by the hydropathy (GRAVY) index using ProtParam tool (http://www.expasy.org/proteomictools/protparam), as described previously [20].

#### Microbial adhesion to hexadecane (MATH)

The microbial adhesion to hexadecane (MATH) assay was employed to evaluate the hydrophobicity of the surface of the bacterial cells obtained from the various fermentations. The method was carried out as described previously [21]. Briefly, the freeze-dried cells were washed with PBS and suspended in 10 mM KH_2_PO_4_ (Sigma) to obtain an OD_600_ ~ 0.8. The pH of the suspension was adjusted to 3 with 1 M HCl. 2 ml of the bacterial cell suspension was then mixed with equal volume of hexadecane (Sigma) in a 10 ml syringe. The mixture was vortexed for 1 min and then left undisturbed for 20 min to allow complete phase separation. After equilibration, the aqueous phase was removed carefully, in order not to disturb the interfacial equilibrium, and the optical density (OD_600_) was measured. The percentage adhesion (% adhesion) was calculated using the following equation:

(1) $$\%\;\text{adhesion to hexadecane}=\left(1-\frac{{\text{A}}_{1}}{{\text{A}}_{0}}\right)\times 100$$

where A_0_ is the initial absorbance (at 600 nm) of the bacterial suspension and A_1_ is the absorbance after 20 min of incubation.

#### Adhesion to Caco-2 cells

The adhesion assay was performed as described previously [14] with slight modifications. More specifically, after addition of the bacterial cells into the wells and incubation for 60 min at 37°C, in 5% CO_2_ and 95% air, the Dulbecco’s phosphate buffered saline (DPBS) (Oxoid) fraction containing unbound bacteria from each well was transferred into a sterile tube. The wells were further washed with 1 ml of DPBS to remove any non-specifically bound bacteria, and the wash added into the same tube. The number of bacteria bound to the Caco-2 cells was determined by subtracting the number of unbound bacteria from the total number of bacteria added to the well. Each adhesion experiment was performed in triplicate.

### Statistical analysis

For statistical comparisons, one-way ANOVA was used (with 95% confidence interval), using SPSS (IBM SPSS Inc.).

## Results

### Bacterial growth

**Figure 1:**
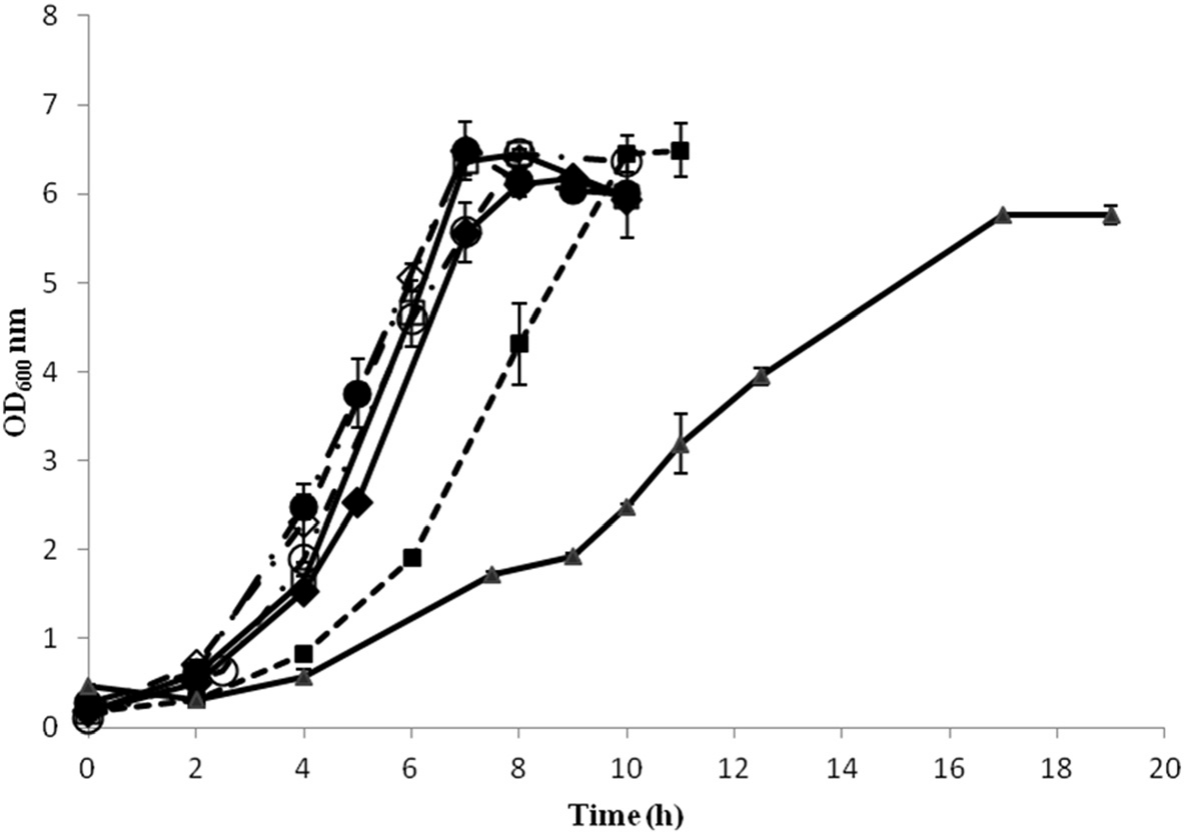
**Growth curve of*L. rhamnosus*GG under different fermentation conditions.** (♦) pH 6.5, 37°C control; (●) uncontrolled pH, 37°C; (□) pH 6, 37°C; (◊) pH 5.5, 37°C; (○) pH 5, 37°C; (■) pH 6.5, 30°C and pH 6.5, (▴) 25°C*.* Error bars represent standard deviations.

Figure 1 depicts the cell growth under the different fermentation conditions. The cells grew well in most conditions, reaching a maximum OD_600_ value between 6 and 7 in about 6 h to 8 h. In the case of the fermentation carried out at 25°C, the maximum OD_600_ was around 5.5. In the case of the fermentations carried out at different pH, the growth rates (μ) were similar ranging between 0.92 h^-1^ and 1.10 h^-1^, with that of pH 5 slightly higher than the rest. In the case of the fermentations carried out at different temperatures, the cells grew slower at 30°C (μ ~ 0.77 h^-1^, stationary phase was reached after 10 h) than in the control fermentation, and considerably slower at 25°C (0.36 h^-1^, stationary phase was reached after 17 h) (Table 1).

**Table 1:** **Growth rates of*L. rhamnosus*GG growing at different fermentation conditions**

**Fermentation**	**Growth rate (h**^**-1**^**)**
37°C, pH 6.5	0.95
37°C, uncontrolled pH	0.92
37°C, pH 6	0.97
37°C, pH 5.5	0.95
37°C, pH 5	1.10
30°C, pH 6.5	0.77
25°C, pH 6.5	0.36

### XPS analysis

The elemental composition of the bacterial surfaces, resulting from integrating the C1s, O1s, N1s, and P2p peaks from the survey spectra, and the functional group compositions are presented in Table 2 (Annex 1 for a representative spectrum, Additional file 1). The nitrogen appeared at a binding energy of 398.59 eV and is attributed to the amine or amide groups of proteins. The phosphorus appeared at a binding energy of 132.15 eV and is attributed to phosphate groups. The XPS peaks corresponding to C and O were analyzed at high resolution and were de-convoluted to assess the contributions from each component. The carbon peak (C1s) was fit into four components: carbon bound only to carbon and hydrogen, Cs(C,H), at 282.7 eV; carbon singly bound to oxygen or nitrogen from ethers, alcohols, amines or amides, Cs (O,N), at 284.1 eV; carbon doubly bonded to oxygen or singly bonded to two oxygen atoms from amides, carbonyls, carboxylates, esters, acetals or hemi-acetals, C=O and O-C-O, at 285.4 eV; and carbon attributable to carboxylic functions, COOR, at 286.7 eV. The oxygen peak (O1s) was best fit with three contributions: oxygen double bonded with carbon or phosphorus from carboxylic acids, carboxylates, esters, carbonyls, amides, or phosphoryl groups, C=O and P=O, at 529.7 eV; and oxygen attributable to hydroxide, phosphate, acetal, or hemiacetal, C-OH, C-O-C, and PsOH, at 530.5 eV. The oxygen peak (O_1s_) was best fit with three contributions: oxygen double bonded with carbon or phosphorus from carboxylic acids, carboxylates, esters, carbonyls, amides, or phosphoryl groups, C=O and P=O, at 529.7 eV; oxygen attributable to hydroxide, phosphate, acetal, or hemiacetal, C-OH, C-O-C, and P-OH, at 530.5 eV; and oxygen attributable to trapped water, at 534.5781 eV [22].

**Table 2:** **XPS analysis of freeze dried*L. rhamnosus*GG cells produced at different fermentation conditions: pH 6.5, 37°C (control); pH uncontrolled, 37°C; pH 6, 37°C; pH 5.5, 37°C; pH 5, 37°C; pH 6.5, 30°C and pH 6.5, 25°C**

	**Control pH 6.5, 37°C**	**Uncontrolled pH 37°C**	**pH 6 37°C**	**pH 5.5 37°C**	**pH 5 37°C**	**30°C pH 6.5**	**25°C pH 6.5**
**Total C**	66.73 ± 2.26	63.70 ± 5.3	65.87 ± 0.7	69.62 ± 4.2	68.81 ± 0.5	71.21 ± 7.4	66.34 ± 1.1
**Total O**	27.58 ± 2.6	30.70 ± 3.3	25.72 ± 0.4	20.60 ± 3.3	24.78 ± 0.3	24.61 ± 0.2	24.61 ± 1.4
**Total N**	4.49 ± 0.4	5.86 ± 0.5	7.58 ± 1.2	9.03 ± 2.6	6.45 ± 0.5	6.88 ± 1.1	8.01 ± 0.4
**Total P**	1.20 ± 0.5		0.83 ± 0.3				1.04 ± 0.3
**O/C**	0.41 ± 0.04	0.48 ± 0.06	0.39 ± 0.01	0.30 ± 0.05	0.36 ± 0.01	0.35 ± 0.03	0.37 ± 0.02
**N/C**	0.07 ± 0.01	0.09 ± 0.01	0.11 ± 0.02	0.13 ± 0.04	0.09 ± 0.01	0.10 ± 0.01	0.12 ± 0.01
**P/C**	0.02 ± 0.01		0.01 ± 0.01				0.02 ± 0.01
**C-(C,H)**	31.68 ± 3.06	46.04 ± 7.52	35.19 ± 2.70	44.13 ± 6.90	31.68 ± 2.19	36.93 ± 0.71	34.82 ± 4.97
**C-(O,N)**	45.79 ± 2.11	37.81 ± 3.62	42.80 ± 1.7	31.20 ± 7.80	46.51 ± 1.38	40.56 ± 0.60	43.9 ± 3.91
**C = O, O-C-O**	17.62 ± 0.81	16.07 ± 2.24	19.11 ± 1.05	19.28 ± 1.35	18.62 ± 0.72	18.25 ± 1.44	18.92 ± 0.97
**COOR**	4.91 ± 3.26	1.26 ± 0.33	2.91 ± 0.27	1.59 ± 0.31	3.21 ± 0.25	4.27 ± 1.54	2.41 ± 0.30
**O-(C,P)**	71.64 ± 2.30	65.74 ± 3.47	68.39 ± 4.20	52.77 ± 0.29	70.94 ± 1.02	56.38 ± 0.69	58.52 ± 6.30
**O = (C,P)**	21.79 ± 2.27	30.48 ± 1.10	4.33 ± 0.12	47.40 ± 6.05	21.53 ± 1.58	37.27 ± 1.80	32.96 ± 4.92
**O-H-O**	6.56 ± 3.75	1.78 ± 0.33	27.21 ± 30.43	4.37 ± 2.21	7.53 ± 0.57	6.36 ± 1.11	3.92 ± 0.11

Results are presented as mean ± SD (standard deviation).

The survey scans revealed that the bacterial cell surface from all the fermentations consisted mainly of C, O and N. Small amounts of P were detected on the surface of the bacteria from the control fermentation (pH 6.5, 37°C), as well as from the fermentations carried out at pH 6 (37°C) and at 25°C (pH 6.5). Compared to the control fermentation, the cells from all the fermentations that were carried out exhibited higher nitrogen to carbon ratios (N/C), whereas the O/C ratios were in some cases slightly lower (pH 5, pH 5.5, pH 6, 30°C and 25°C), or slightly higher (uncontrolled pH). In respect to the functional components, the cells from the control fermentation and the fermentation carried out at pH 5 showed a higher concentration of O-(C,P) and a lower concentration of O=(C,P) compared to the rest of the fermentations. Although the differences were small, the cells from the control fermentation and the fermentation carried out at pH 5 showed higher C-(N,O) concentrations compared to the rest.

### FT-IR analysis

**Figure 2:**
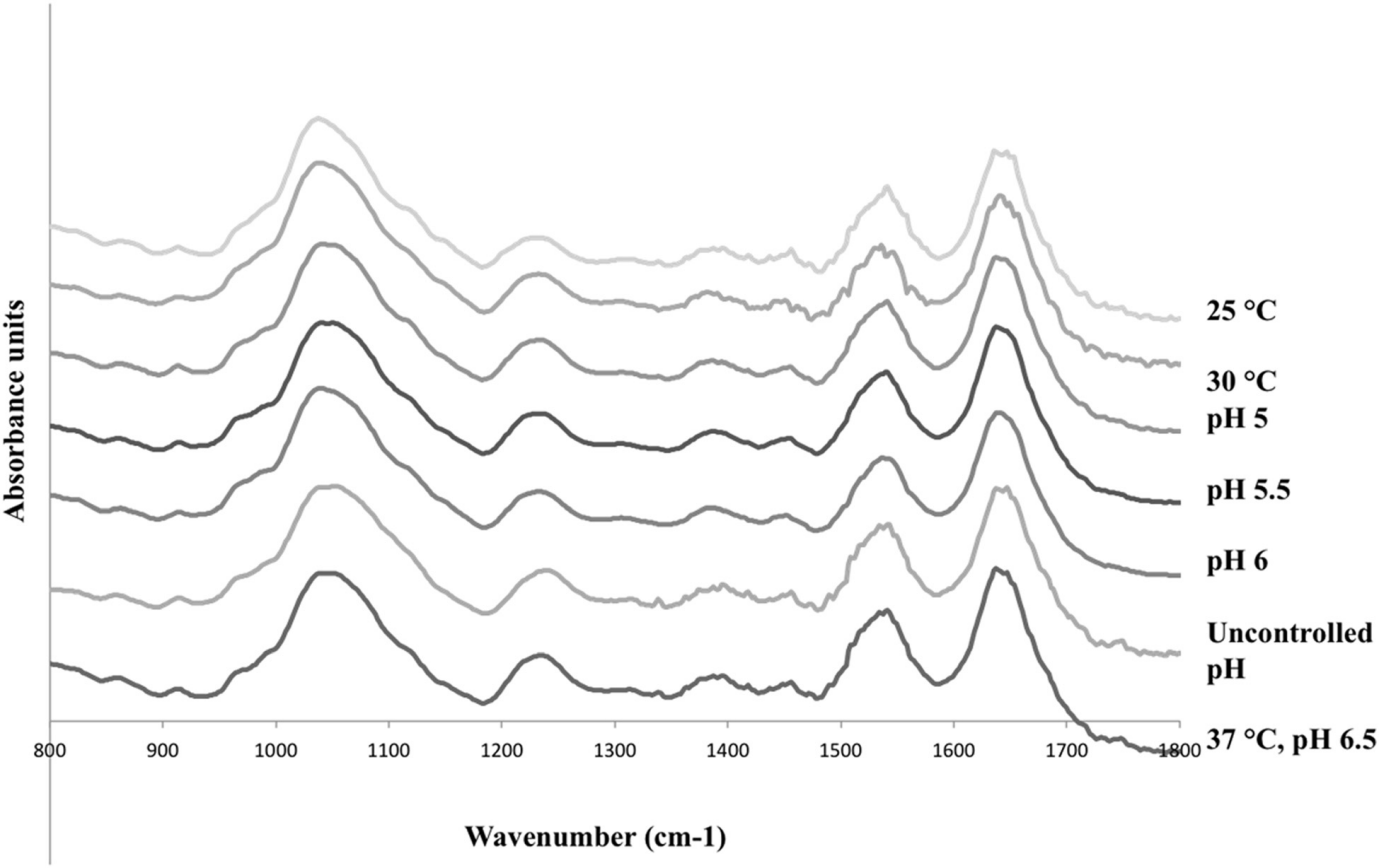
**FT-IR spectra of*L. rhamnosus*GG produced at different fermentation conditions: pH 6.5, 37°C (control); pH uncontrolled, 37°C; pH 6, 37°C; pH 5.5, 37°C; pH 5, 37°C; pH 6.5, 30°C and pH 6.5, 25°C.** The spectra are the average of scans from two fermentations.

Figure 2 presents the FT-IR spectra of the bacterial cells grown in the different fermentation conditions. The absorption band assignments corresponding to the functional groups of macromolecules are summarised in Table 3. Among the samples, differences were observed in the carbohydrate region (1200 – 950 cm^-1^) of the spectra, and also in the areas corresponding to the stretching of phosphate groups (~1225 cm^-1^) and of the C=O and C-N groups in the amide I and II bands, respectively. In particular, in the cells from the fermentations carried out at 25°C and 30°C, peak shifts were observed in the carbohydrate region, whereas in the cells from the uncontrolled pH fermentation, a peak shift was observed in the phosphate region.

**Table 3:** **Functional group assignment of FT-IR spectra (adapted from**[17]**)**

**Wave number (cm**^**-**^**1)**	**Functional group assignment**
1739-1725	stretching CdO of ester functional groups from membrane lipids and fatty acids; stretching CdO of carboxylic acoid
1647	stretching CdO in amides (amide I band); bending -NH and -NH2 of amines
1548	N-H bending and C-N stretching in amides (amide II band); bending -NH and -NH2 of amines
1402	symmetric stretching for deprotonated COO- group
1453	bending CH2/CH3 (scissoring)
1384	symmetric stretching of COO-; bending CH2/CH3
1305	vibration C-N from amides
1300-1250	vibrations of C-O from esters or carboxylic acids
1262	vibrations of -COOH and C-O-H; double bond stretching of > PdO of general phosphoryl groups and phosphodiester of nucleic acids
1225	stretching of PdO in phosphodiester of nucleic acids
1225	stretching CdO in phosphates
1200-950	asymmetric and symmetric stretching of PO2- and P(OH)2 in phosphate; vibration of C-OH, C-O-C, and C-C of polysaccharides
1085	stretching PdO of phosphodiester, phosphorylated proteins, or polyphosphate products
976	symmetric stretching vibration of phosphoryl groups

Principle Component Analysis (PCA) was performed on the data (see Additional file 2: Figure S1). The PCA showed that the 25°C 6.5 pH sample clustered separate from the rest and control conditions were significantly different from the rest of the conditions. Though the subtle differences observed were not picked in the PCA analysis and were not statistically significant, they possibly can have an effect on the surface properties.

### Surface protein profile

**Figure 3:**
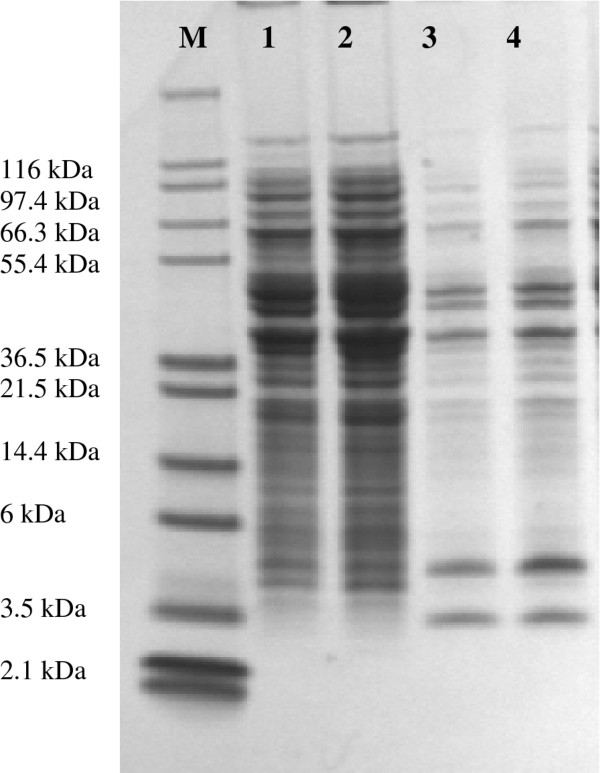
**Representative SDS page gel image of potential proteins on the surface of*L. rhamnosus*GG.** Lanes: M: protein marker (Mark12); 1,2: control fermentation (duplicate samples); 3,4: uncontrolled fermentation (duplicate samples).

The SDS PAGE analysis showed similar protein profiles between the different fermentation conditions. Figure 3 depicts a representative gel of the surface proteins of the control fermentation (37°C, pH 6.5) and the uncontrolled fermentation (37°C, uncontrolled pH). In total, 82 proteins were identified at the surface of the cells by proteomic analysis (Annex 2, Additional file 3), with the majority of them being common for all fermentations. Table 4 lists the most important surface-associated proteins that were identified on the surface of the cells, and which were previously shown or suggested to be involved in the adhesion of lactobacilli or other Gram positive bacteria to various cell lines, mucus, and extracellular matrix components. Four proteins that have been commonly associated with the adhesion of various lactobacilli to the gastrointestinal mucosa, namely α-enolase, elongation factor Tu (EF-Tu), glyceraldehyde-3-phosphate dehydrogenase (GAPDH), and GroES chaperonin were identified on the cell surface. The GRAVY index suggested that all these proteins had a hydrophilic character.

**Table 4:** **List of proteins identified on the surface of*L. rhamnosus*GG that have been associated with adhesion to cell lines, mucin and extracellular matrix components**

**Protein names**	**Strain**	**Site of adhesion**	**Reference**	**Mass kDa**
10 kDa chaperonin	*Staph. aureus*	NT^*^	[50]	10
30S ribosomal protein	*L. rhamnosus* GG	NT	[49]	47
50S ribosomal protein	*Strep. pyogenes*	NT	[51]	12
60 kDa chaperonin *(*GroEL*)*	*L. johnsonii*	Human intestinal cells and mucus	[52]	57
Aminopeptidase C	*L. rhamnosus* GG	NT	[49,53]	51
Cell division protein	*Staph. aureous*	NT	[50]	78
Elongation factor Tu	*L. plantarum*	Porcine mucin; Caco-2 cells	[16,42]	44
Endopeptidase O	*L. rhamnosus* GG	NT	[49]	73
Enolase	*L. plantarum*	Fibronectin	[54]	47
GAPDH	*L. plantarum*	Porcine mucin; Caco-2 cells	[16,42,49]	37
GMP synthase	*L. plantarum*	Porcine mucin	[16]	58
Phosphoglycerate kinase	*L. rhamnosus* GG	NT	[49]	42
Trigger factor	*L. plantarum*	Porcine mucin	[16]	50
Triosephosphate isomerase	*L. rhamnosus* GG	NT	[49]	27

NT*: not tested.

### Cell surface hydrophobicity

**Figure 4:**
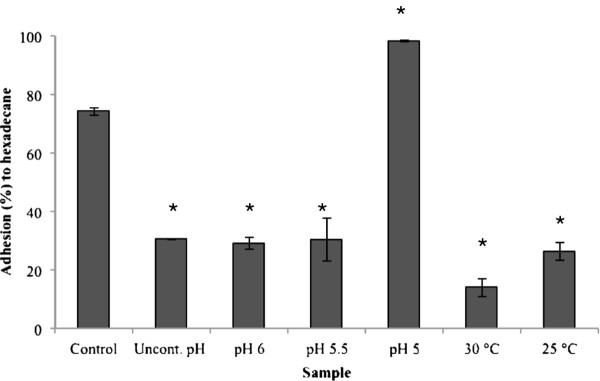
**% Adhesion to hexadecane of*L. rhamnosus*GG cells produced at different fermentation conditions: pH 6.5, 37°C (control); pH uncontrolled, 37°C; pH 6, 37°C; pH 5.5, 37°C; pH 5, 37°C; pH 6.5, 30°C and pH 6.5, 25°C.** Error bars represent standard deviation. The star (*) represents statistical significant difference compared to the control fermentation (p < 0.05).

Figure 4 presents the results from the MATH assay. It can be observed that the highest hydrophobicity value was obtained for the cells grown at pH 5, which showed almost 100% adhesion to hexadecane, and significantly (p < 0.05) higher than the control fermentation. The cells from all the other fermentations were significantly (p < 0.05) less hydrophobic compared to the control fermentation.

### Adhesion to Caco-2 cells

**Figure 5:**
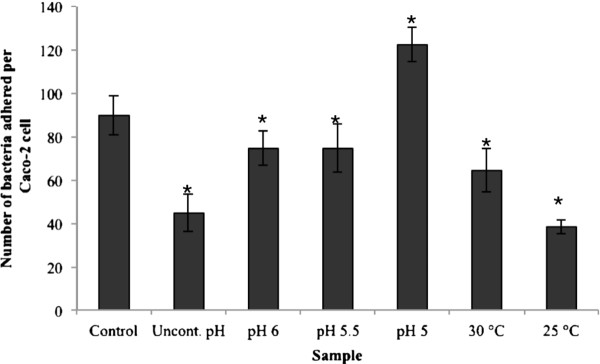
**Adhesion of*L. rhamnosus*GG to Caco-2 cells.** Error bars represent standard deviation. The star (*) represents statistical significant difference compared to the control fermentation (p < 0.05).

Figure 5 presents the results from the adhesion of the bacterial cells, obtained from the different fermentations, to Caco-2 cells. The maximum adhesion value, almost 120 bacteria per Caco-2 cell, was obtained for the cells from the fermentation carried out at pH 5, and was followed by the control fermentation. The bacterial cells from the rest of the fermentations were significantly (p < 0.05) less adhesive than the cells from the control fermentation. Similarly to the hydrophobicity trend, the bacterial cells that were grown at suboptimal temperatures (25°C, 30°C) were significantly (p < 0.05) less adhesive than the bacterial cells grown at sub-optimal pH (5, 5.5, 6).

## Discussion

The aim of this work was to evaluate the effect of the fermentation conditions, and in particular the pH and temperature, on the surface properties of *L. rhamnosus* GG and its adhesion to Caco-2 cells. The influence of the production process on the surface characteristics and adhesion abilities of probiotic lactobacilli is a relatively unexplored area. Recent studies from our research group and other published works have shown that the incubation time and the composition of the fermentation medium are important factors [7,14,23], whereas studies focusing on the downstream processing, e.g. drying of probiotics, have shown that drying affects the physicochemical surface properties of *Lactobacillus* cells [24,25]. Identifying possible ways to control the surface properties of the bacterial cells is very important in order to produce cells with enhanced functionality. This is the first study of its kind that looks at the fermentation and processing part and tries to relate the physical and biochemical properties of the bacterial cells to find answers related to the functional aspects of the probiotics, such as adhesion to the human GI tract.

It can be deduced from the growth curves in Figure 1 that carrying out the fermentation at suboptimal temperatures (i.e. 25°C and 30°C) resulted in lower growth rates, and in the case of the 25°C to a lower final cell density too, compared to 37°C. In contrast, the pH of the fermentation, within the range studied, did not seem to affect considerably the growth rate, nor the final cell density. The literature has shown that the pH and temperature influence the growth behaviour of lactobacilli and the final yield obtained, although substantial differences can be observed, depending on the strain. It has also been reported that a lower pH of fermentation, for example pH 5 compared to pH of around 6, is likely to result to the production of physiologically more robust cells [26], which are able to survive better freezing and frozen storage [12], freeze drying [27] and acid stress [28]. In the case of temperature, it was also suggested that suboptimal conditions of growth, for example 30°C compared to 37°C, produced cells that were better able to survive freezing and frozen storage. A link was also shown to exist between the membrane fatty acid composition and increased cryotolerance [12]. The conditions used in this study for the control fermentation, i.e. 37°C and pH 6.5, are typical for *Lactobacillus* growth and reflect the optimal conditions for achieving high cell yields, while the fermentation medium used is typical of a growth medium used for large production, and was optimised in a previous study in terms of cell growth and cell survival upon freeze drying [10]. The rationale behind selecting pH and temperature values lower than the optimal was the increased robustness and improved technological properties observed in previous studies, which were most likely associated with compositional and conformational changes taking place at the cell membrane. Thus, it could be likely that important changes were also taking place at the surface of the cells when growing the cells at such conditions, which would affect their adhesion properties.

The adhesion levels of the cells from the different fermentations ranged from 40 to 120 bacteria per Caco-2 cell (Figure 5), which is within the range of previous reports [14,29,30]. The main source of knowledge about the adhesive properties of lactobacilli is from studies using *in vitro* model systems, although *in vivo*/*ex vivo* studies have also been used to a much lesser extent [31]. Various studies have shown a good correlation between *in vitro* adhesion, using for example Caco-2 and HT29 cell lines, and *in vivo* adhesion based on the results from human intervention studies [32,33]. Tissue culture cells, such as Caco-2, HT-29 and HT-29 MXT cells, are commonly used for *in vitro* adhesion studies [34-36]. Caco-2 cells exhibit many properties of the small intestine as they form a polarised monolayer of differentiated columnar absorptive cells expressing a brush border [37]. In the present study, with the exception of the cells from the fermentation carried out at pH 5, the rest of the cells were significantly less adhesive to Caco-2 cells compared to the cells from the control fermentation (p < 0.05).

The lowest adhesion to Caco-2 cells was observed for the cells from the 25°C and uncontrolled pH fermentation, which had a higher total N content and N/C ratio as compared to the control sample. The cells from the 25°C fermentation showed also a slightly lower total O content and O/C ratio compared to the control fermentation. In the same way, the cells from the 30°C were characterised by a lower adhesion value and a higher total N content and N/C ratio (0.10) than the control fermentation. The above suggest that the exposure of proteins at the surface of the cells was higher than in the case of the control fermentation, whereas the exposure of carbohydrates and phosphate-containing compounds was probably lower. Consequently, it could be deduced that a cell surface richer in proteins resulted in a lower adhesion to Caco-2 cells, a conclusion that has also been suggested by our previous published studies [14]. No differences were observed between the concentrations of various functional groups, especially the C-(O,N), C=O and O-C-O groups, which could correspond to carbohydrates. On the other hand, a considerable increase in the case of the O = (C,P) group for the uncontrolled pH, pH 5.5, 25°C and 30°C fermentations was observed compared to the control fermentation. This could potentially suggest an increase in the concentration of amide bonds, and thus of proteins. The results obtained from the XPS analysis coincided with the FT-IR results, although it is difficult to quantitatively correlate these. The FT-IR data showed a peak shift in the carbohydrate region of the spectra (1200 – 950 cm^-1^), as well as a number of peaks that emerged in the amide I and II regions, which could suggest that conformational changes took place in the cell surface components. Principle Component Analysis (PCA) was performed on the FTIR data. Though the subtle differences observed in the peak shift were not picked in the PCA analysis and were not statistically significant. These changes along with other compositional and conformational modifications of the bacterial surface might have an effect on the net surface properties such as adhesion to Caco-2 cells. Interestingly, the hydrophobicity of the cells from the 25°C and 30°C fermentations was drastically reduced compared to the control, which is difficult to explain, as the general consensus is that hydrophilic cells are most likely covered by carbohydrates [7,38,39]. However, this depends on the strain, and it most likely applies for *Lactobacillus* strains that contain the highly hydrophobic S-layer proteins, meaning that fermentation conditions that do not favour the expression of the S-layer proteins on the surface result in more hydrophilic cells. However, *L. rhamnosus* GG does not contain an S-layer. In addition, calculation of the GRAVY index for the key surface proteins that were identified by proteomic analysis, and which are listed in Table 4, indicated that they were all hydrophilic. This could thus explain the observed decrease in the hydrophobicity for these particular samples.

The cells from the fermentations carried out at pH 6, pH 5.5, and with no pH control, exhibited significantly (p < 0.05) lower adhesion to Caco-2 cells compared to the cells from the control fermentation. The atomic ratios and the concentrations of the functional groups obtained from XPS analysis, were similar to those for the 25°C and 30°C fermentations, suggesting that the cells had a protein-rich surface with very low amounts of phosphate-containing compounds. These observations were also in agreement with the hydrophobicity values, which were considerably lower than in the case of the control fermentation. Regarding the fermentation carried out at pH 5, the cells showed higher adhesion than the cells from the control fermentation, and very high hydrophobicity as compared to the control (p < 0.05). It is interesting to note that the XPS data in this case were more similar to the data from the control fermentation rather than to the other fermentations, and more specifically the total N and O content. Also, the concentrations of the C-(O,N), C=O and O-C-O groups were all similar, suggesting a similar surface composition to the cells from the control fermentation. The differences in adhesion between the cells from the pH 5 fermentation and the control fermentation could be either due to differences in the surface composition, in particular carbohydrates, which were not picked up by the XPS analysis, or to differences in the levels or profiles of the surface proteins. However, proteomic analysis indicated no differences in the protein profiles between all the fermentation samples, although quantitative analysis was not conducted; such work is planned for the future. In summary, all the samples contained proteins that have been associated with the adhesion of various lactobacilli. Table 4 enlists the proteins identified on the bacterial cell surface, their target site and molecular mass, as reported in previous studies. Among these, α-enolase, elongation factor Tu (EF-Tu), glyceraldehyde-3-phosphate dehydrogenase (GAPDH), and GroES chaperonin stand out in terms of likely importance. More specifically, α-enolase has been shown to be involved in the adhesion of *L. johnsonii* and *L. crispatus* to extracellular matrix components [40,41], EF-Tu in the adhesion of *L. plantarum*[42] and *L. johnsonii*[43] to intestinal cells, GroEL in the adhesion of probiotics and pathogens to the gastrointestinal mucosa [44], and GAPDH in the adhesion of *L. plantarum* to Caco-2 cells [45]. It is interesting however to note that the association of enolase and GAPDH with the cell wall of *L. crispatus* has been shown to be pH dependent, with the strongest association being at pH 5 [46]. In accordance with this, the high levels of adhesion observed for the grown at pH 5, could be attributed to the stronger association of these two proteins with the cell wall of *L. rhamnosus* GG, although this is a hypothesis that needs to be investigated further.

A pili containing human mucus binding protein has been identified in *L. rhamnosus* GG, which could explain how this strain may persist in the host and compete with pathogens for residence sites in the human intestinal tract [47]. The study reported that the pili were required for the adhesion of the bacteria to the host and suggested a possible role of pili in other probiotic effects as well. Another study from the same group reported a number of pilin subunits in the same strain and showed that they play a role in the adhesion of bacteria to the intestinal mucus [48]. In the present study the pili protein was detected, however not at significant levels, therefore it was not included in the analysis. A similar result was also reported in another study on the surface proteins of *L. rhamnosus* GG [49]. The reasons for this are likely to be differences in the extraction process or the growth conditions.

## Conclusions

The temperature and the pH of the fermentation influenced the surface composition, hydrophobicity and the levels of adhesion of *L. rhamnosus* GG to Caco-2 cells. It was deduced from the data that a protein rich surface reduced the adhesion ability of the cells. The study showed that 37 C growth temperature and pH 6.5 are the best conditions of production of LGG cells with high adhesion ability. Use of suboptimal pH 5 stress also seemed to have a positive effect on bacterial adhesion. Therefore, manipulating the fermentation conditions towards the production of cells with carbohydrate rich surfaces is likely to result to the production of more adhesive bacterial cells.

## Competing interests

The authors declare that they have no competing interests.

## Authors’ contributions

GD carried out the experimental work and drafted the manuscript, EK helped in carrying out the proteomic work, CB kindly gave advice on the surface analysis and provided the facilities for proteomic, XPS and FTIR work, CRH performed the XPS work and DC provided the overall guidance and supervision of the project.

## Supplementary Material

Additional file 1 XPS spectrum of pH uncontrolled Fermentation. Click here for file

Additional file 2 Figure S1. Principle Component Analysis. Click here for file

Additional file 3 **List of proteins detected on the surface of*L. rhamnosus *GG. ** Click here for file
