# Supplementary figures and images for: Influence of fermentation conditions on the surface properties and adhesion of Lactobacillus rhamnosus GG
Source: Microb Cell Fact. 2012 Aug 29;11:116. doi: 10.1186/1475-2859-11-116 (PMC3441878; doi:10.1186/1475-2859-11-116)

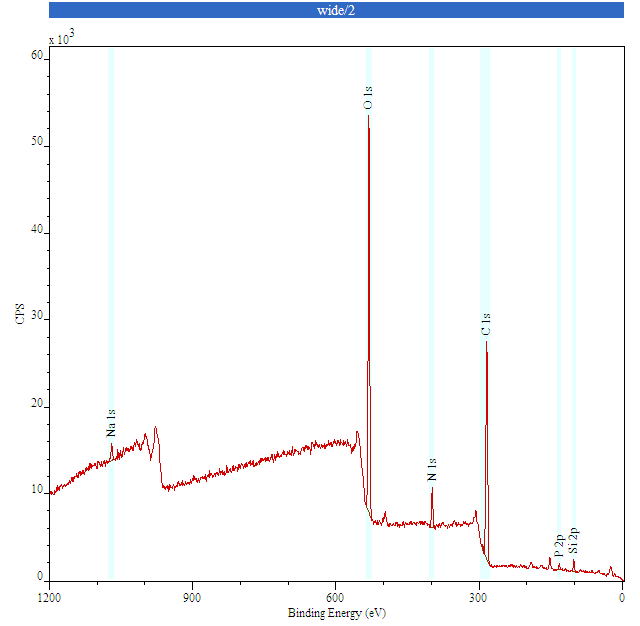

Supplement: Additional file 1 — XPS spectrum of pH uncontrolled Fermentation. [file 1475-2859-11-116-S1.bmp]
