# Supplementary material for: Influence of fermentation conditions on the surface properties and adhesion of Lactobacillus rhamnosus GG
Source: Microb Cell Fact. 2012 Aug 29;11:116. doi: 10.1186/1475-2859-11-116 (PMC3441878; doi:10.1186/1475-2859-11-116)
Supplement: Additional file 2 — Figure S1. Principle Component Analysis. [file 1475-2859-11-116-S2.doc]

**Additional File 1 Principle Component Analysis**


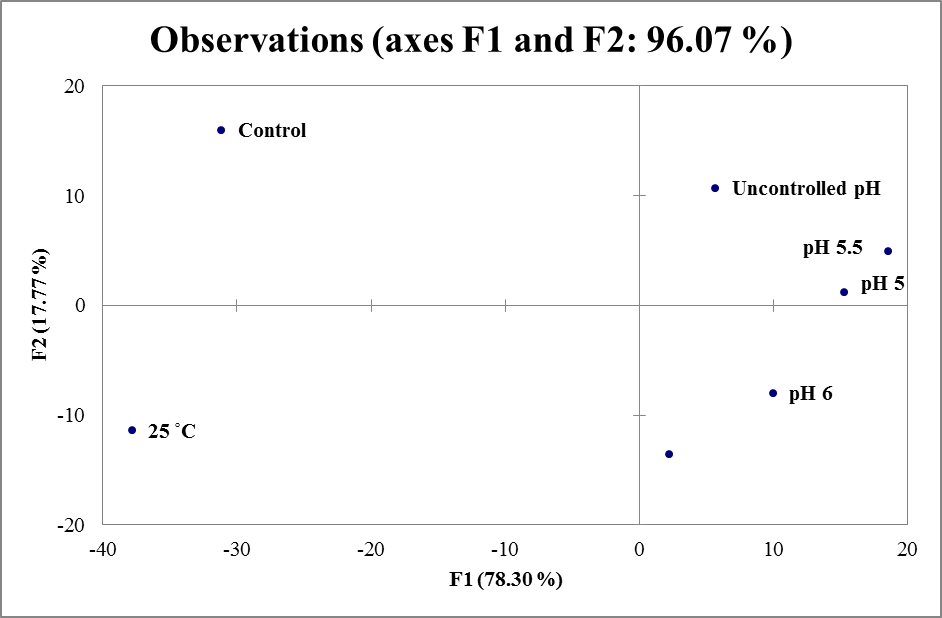


**30 ˚C**

Figure I principal component analysis of ATR-FTIR spectra of *L. rhamnosus* GG produced at different fermentation conditions: pH 6.5, 37 °C (control); pH uncontrolled, 37 **°**C; pH 6, 37 **°**C; pH 5.5, 37 **°**C; pH 5, 37 **°**C; pH 6.5, 30 **°**C and pH 6.5, 25 **°**C.
