# Supplementary material for: Influence of fermentation conditions on the surface properties and adhesion of Lactobacillus rhamnosus GG
Source: Microb Cell Fact. 2012 Aug 29;11:116. doi: 10.1186/1475-2859-11-116 (PMC3441878; doi:10.1186/1475-2859-11-116)
Supplement: Additional file 3 — List of proteins detected on the surface ofL. rhamnosus GG. [file 1475-2859-11-116-S3.doc]

**Annex 2**

| **List of proteins detected on the surface of *L. rhamnosus* GG** |
| --- |
| 1. 2,3-bisphosphoglycerate-dependent phosphoglycerate mutase |
| 1. 30S ribosomal protein |
| 1. 50S ribosomal protein |
| 1. 3-carboxymuconate cyclase |
| 1. 60 kDa chaperonin |
| 1. 6-phosphogluconate dehydrogenase, decarboxylating |
| 1. Adenylosuccinate lyase |
| 1. Adenylosuccinate synthetase |
| 1. Aldo/keto reductase |
| 1. Aminopeptidase C |
| 1. Aspartate carbamoyltransferase |
| 1. Aspartate/tyrosine/aromatic aminotransferase |
| 1. Aspartyl/glutamyl-tRNA amidotransferase A subunit |
| 1. ATP-dependent nuclease, subunit B |
| 1. Bifunctional purine biosynthesis protein purH |
| 1. Branched-chain-amino-acid aminotransferase |
| 1. Cell division protein, signal recognition particle FtsY |
| 1. Cysteine synthase |
| 1. Deoxyribose-phosphate aldolase |
| 1. Dihydroorotase |
| 1. D-lactate dehydrogenase |
| 1. D-fructose-6-phosphate amidotransferase |
| 1. DNA polymerase III, beta subunit |
| 1. dTDP-4-dehydrorhamnose reductase |
| 1. dTDP-glucose 4,6-dehydratase |
| 1. Elongation factor G |
| 1. Elongation factor Ts |
| 1. Elongation factor Tu |
| 1. Endopeptidase O |
| 1. Enolase |
| 1. Fructose-bisphosphate aldolase |
| 1. Galactose mutarotase (Galactose mutarotase enzyme, lacX protein) |
| 1. Glucosamine--fructose-6-phosphate aminotransferase, isomerizing |
| 1. Glucose-1-phosphate thymidylyltransferase |
| 1. Glucose-6-phosphate isomerase |
| 1. Glyceraldehyde-3-phosphate dehydrogenase |
| 1. GMP synthase |
| 1. GTP-binding protein (GTP-binding protein, HflX subfamily) |
| 1. GTP-binding protein YchF |
| 1. GTP-binding protein, HflX subfamily |
| 1. Heat-inducible transcription repressor (Heat-inducible transcription repressor hrcA) |
| 1. Inorganic pyrophosphatase (Manganese-dependent inorganic pyrophosphatase) |
| 1. Integrase |
| 1. L-lactate dehydrogenase 2. Lipoate- protein ligase A |
| 1. Manganese-dependent inorganic pyrophosphatase |
| 1. Mannitol-1-phosphate 5-dehydrogenase |
| 1. MccC family protein (Microcin C7 resistance protein) |
| 1. NAD(FAD)-dependent dehydrogenase (NADH peroxidase) |
| 1. NADH peroxidase |
| 1. Nucleotide-binding protein (Universal stress protein, UspA family) |
| 1. Oligoendopeptidase F |
| 1. Orotate phosphoribosyltransferase |
| 1. Oxidoreductase |
| 1. Oxidoreductase, aldo/keto reductase family |
| 1. Putative GTPase |
| 1. Seryl-tRNA synthetase |
| 1. Single-stranded DNA-binding protein |
| 1. SSU/30S ribosomal protein S1P |
| 1. Peptide chain release factor (Peptide chain release factor 1) |
| 1. Peptidyl-prolyl cis-trans isomerase |
| 1. Phosphate acetyltransferase |
| 1. Phosphoenolpyruvate-protein phosphotransferase |
| 1. Phosphoglucomutase |
| 1. Phosphoglycerate kinase |
| 1. Phosphoribosylamine--glycine ligase (Phosphoribosylamine-glycine ligase) |
| 1. Phosphoribosylaminoimidazole carboxylase, ATPase subunit |
| 1. Phosphoribosylaminoimidazole-succinocarboxamide synthase |
| 1. Phosphoribosylformylglycinamidine synthase I |
| 1. Protein Translation Elongation Factor G (EF-G) |
| 1. PTS system enzyme I (Phosphoenolpyruvate-protein phosphotransferase) |
| 1. Putative phosphosugar isomerase (Tagatose-6-phosphate ketose/aldose isomerase) |
| 1. Pyrimidine operon regulator |
| 1. Pyruvate kinase |
| 1. Ribose-5-phosphate isomerase A |
| 1. S-adenosylmethionine synthetase |
| 1. Tagatose 1,6-diphosphate aldolase |
| 1. Tagatose-6-phosphate ketose/aldose isomerase |
| 1. Trigger factor |
| 1. Triosephosphate isomerase |
| 1. Universal stress protein, UspA family |
| 1. Xanthine phosphoribosyltransferase |
